# The Proposed London Hospital for the Insane

**Published:** 1908-04-04

**Authors:** 


### Mental Diseases.

### THE PROPOSED LONDON HOSPITAL FOR THE INSANE.

Last year we published a series of articles upon the present unsatisfactory state of medical study and treatment of lunacy, and pointed out that since the Royal Hospital of Bethlem and the other registered hospitals have neglected their mission, and the rate-supported asylums cannot be expected to take their place as centres of research and teaching, the need is urgent for an institution for the study and treatment of mental diseases, carried on upon the lines of the ordinary charitable general hospital, with a visiting staff of physicians of high eminence, resident staff, laboratories, and appliances for research, and a school for clinical teaching attached. Until these conditions are fulfilled, there is little prospect of the knowledge of insanity being levelled up to the stage that knowledge of bodily disease has attained. It is with great pleasure that we learn that the ideal we set before our readers is likely to be realised.

Dr. Maudsley, whose great reputation was made during the boyhood of men now passing middle life, has not yet earned the title of an old man, and his mind is as vigorous and alert, and his interest in the studies to which his life has been devoted, is as keen as ever. Recognising, as every wide-minded man must recognise, the deficiencies in the study and teaching of psychiatry that have been exposed in The Hospital, and being, moreover, a man of great wealth, and of what should, but does not always, go with great wealth, great public spirit, he has offered to the London County Council the munificent gift of £30,000 "towards the cost of the establishment in London of a fitly-equipped hospital for mental diseases"; and the gift has been accepted by the Council.

At the meeting at which the gift was formally offered and accepted, Sir Melvill Beachcroft expressed a "mild regret" that the offer had not been addressed to his Majesty's Government, but it is difficult to see under what department of the Government the money could have been appropriately administered, and it is still more difficult to suppose that, in the present state of the national finances, the very large sum of money necessary to complete the scheme could or would have been found. Though there are features of administration by the County Council of London that are undesirable, and though a more ideal arrangement would have been the establishment of a hospital altogether on the lines of the great general hospitals, administered by a Council or Board of Governors of its own, whose members would have no constituents to consider, no axes to grind, no considerations to keep in view except that of efficiency, yet the advantages of administration by the County Council must be acknowledged to be very great. It has a body—its Asylums Committee—already familiar with the main problems to be grappled with, and consisting of experts in the organisation and administration of similar institutions; it has the command of unlimited funds; it has on the whole the support and confidence of public opinion; and it has already for many years had the matter under its consideration.

The difficulty of a rate-supported institution applying its funds to research and clinical teaching, a difficulty inherent in the source of the funds applicable to its support, was pointed out in our columns. This difficulty may now, owing to the munificence of Dr. Maudsley, be surmounted. If the County Council build and support the hospital, they would be devoting their funds to an object which all would admit to be legitimately within their functions. Thus the gift of Dr. Maudsley would be available for the establishment of that part of the scheme to which the application of public funds might be considered questionable—the creation of a clinical school in close association with the hospital—a school in which research could be undertaken, in which laboratories would be furnished, which would become a centre of post-graduate study, to which the élite of the younger graduates of the metropolis would be attracted; and from which could be sent out men thoroughly trained and equipped to serve in the asylums throughout the country. The knowledge that such men would be available would of itself go far to displace the qualifications now required for such posts—skill at cricket and football, taste and proficiency in music, and so forth. Moreover, the very existence of such a standard as the new hospital would set, would revolutionise the tone of thought and the objects of endeavour in all the asylums in the country.

The assumption by the London County Council of the administration of this great and important scheme is to some extent a guarantee that it will not be worked in the interests of any one school, or for the advantage of any particular group or clique. This is a danger that has to be guarded against. It would be as great a mistake to staff the new hospital exclusively with neurologists as to staff it exclusively with alienists. All sides and all schools of thought should be represented. Everything will depend on the personnel of the new institution, and it is to be hoped that the choice, when the choice comes to be made, will be thoroughly eclectic.

The approximate cost of the whole scheme—site, buildings and equipment for a hospital of 100 beds, is estimated by the Asylums Committee of the Council at £60,000, the balance of which, after using the donation of £30,000, would be provided by the Council, which would maintain the institution when completed. The committee, in their report recommending the acceptance of the offer, state that the most forcible argument for such an institution from the point of view of the patient appeared to them to lie in the fact that it would provide opportunity for individual treatment and close personal attention, so important in the early stages of mental disorder. The committee think that an out-patients' department where persons in the incipient stages of mental disorder could be taken for advice and treatment, might, in many cases, avoid altogether the necessity of certification and restraint.

